# Case Report: Sequential blastocyst transfer for women with unexplained recurrent implantation failure: a case series

**DOI:** 10.3389/frph.2026.1942865

**Published:** 2026-10-07

**Authors:** Ping Yin, Yuanyuan Wu, Min Jiang, Yan Li

**Affiliations:** 1 Medical Affairs, Guangzhou Kapok Medical Investment Co., Ltd., Yuncheng/Guangzhou, China; 2 Reproductive Center, Reproductive and Genetic Hospital of Kapok, Qionghai, Hainan, China

**Keywords:** blastocyst transfer, frozen embryo transfer, IVF, sequential embryo transfer, unexplained recurrent implantation failure

## Abstract

**Background:**

Repeated implantation failure (RIF) is a frustrating situation for patients and medical physicians. In spite of extensive research, the underlying mechanisms of RIF are not fully understood. Endometrial receptivity is considered a critical factor for embryo implantation, particularly in patients with unexplained RIF who have usable embryos. Sequential embryo transfer (ET) has been demonstrated to improve clinical pregnancy rates in RIF patients by optimizing embryo–endometrium synchronization and interaction. In this study, we propose a strategy of sequential blastocyst transfer within a single frozen embryo transfer cycle to improve *in vitro* fertilization (IVF) outcomes in unexplained RIF patients who have failed conventional sequential cleavage/blastocyst ET.

**Case presentation:**

Three women under 40 years of age had undergone repeated failed embryo transfer cycles in our hospital. Based on ESHRE recommendations for RIF investigation, no significant causative or contributory factors were identified, apart from mild intrauterine adhesion or chronic endometritis, treated before embryo transfer. Further embryo transfers still failed, even with regular sequential cleavage/blastocyst transfer. However, all three patients achieved positive clinical pregnancies with sequential blastocyst embryo transfer. Two of the patients had live births and the remaining patient experienced an early spontaneous miscarriage for which a pathogenic deletion of chromosome 21 was identified.

**Conclusion:**

Repeated implantation failure is a complex, challenging, and burdensome condition, which causes considerable psychological and physical suffering for patients and reproductive specialists. Sequential blastocyst transfer is a potential option for patients with unexplained RIF undergoing IVF.

## 1 Introduction

Repeated implantation failure (RIF) is a difficult-to-manage clinical dilemma, affecting 10%–15% of patients undergoing *in vitro* fertilization-embryo transfer (IVF-ET) (1). Implantation (2) is the process where a zona-free blastocyst attaches to and penetrates the endometrium or tissue outside the uterine cavity. This process starts 5–7 days after fertilization, usually resulting in the formation of a gestational sac. Successful implantation depends on a complex interaction of multiple factors (1, 3) including, but not limited to, maternal factors such as age, endometrium and uterine conditions, endometrium preparation regimen, and endometrial receptivity; embryo-related factors such as euploidy status, morphology, and developmental potential; paternal factors such as genetic disorders; laboratory and clinical practice performances; and the molecular mechanism of embryo–endometrium crosstalk during implantation, which has not been fully investigated.

Implantation failure occurs when a successful clinical pregnancy is not achieved with good-quality embryo transfer (4). RIF is commonly considered when more than three embryo transfer attempts have failed with a number of good-quality embryos (5), which should be defined in an individual approach for clinical purposes by estimating the chance of achieving a positive pregnancy and setting a threshold for the cumulative chance of successful implantation to proceed to a further ET or investigation according to ESHRE working group recommends (6). RIF is a distinct clinical phenomenon in assisted reproductive treatment (ART) that encompasses a highly heterogeneous patient population. It is a big challenge for patients and physicians. RIF not only imposes severe psychological and financial burdens on affected patients and their families but also causes reproductive professionals distress and frustration (7, 8). Therefore, individualized treatment strategies should be carefully considered for subsequent transfer attempts.

For couples who have completed a standard fertility workup, the cumulative probability of achieving a successful clinical pregnancy declines after two and more ET cycles. Sometimes, no apparent causative factors are discovered after recommended RIF investigations, leading to the following questions. What are we going to do with the remaining embryos next? What different interventions may yield a different outcome? Dr. Paulson proposed a mathematical formula for guidance: embryo implantation (EI) = embryo quality (EQ) × endometrial receptivity (ER) × transfer efficiency (TE) (9). If TE is not a problem, EQ serves as the rate-limiting step of embryo implantation; however, the quality of cryopreserved embryos cannot be altered. Although, some argue that endometrial receptivity is unlikely to be the primary cause of implantation failure—provided a normal cavity, right dosage and duration of estrogen and progesterone, and adequate endometrium thickness are documented—endometrial receptivity remains very complex. Some influencing factors and underlying mechanisms have been identified in relation to RIF.

Embryo–endometrial synchronization and crosstalk are crucial for implantation (10). However, the process is complex and not yet fully understood. A study (11) showed that the displaced window of implantation (WOI) accounts for at least 25% of RIF incidents. Blastocyst transfer (12) is generally preferred for patients with failed transfers due to its superior synchronization ability with endometrial receptivity, higher implantation rates, and better embryo selection. Sequential embryo transfer (13)—in which one cleavage-stage embryo is transferred on Day 3 and one blastocyst on Day 5—represents an alternative strategy aimed at optimizing the interaction between the embryo and endometrium, thereby increasing the likelihood of successful implantation and pregnancy. Pregnancy rates of sequential ET are better than those of conventional cleavage-stage or blastocyst-only transfer in RIF patients (14–16).

For patients with unexplained RIF who continue to experience implantation failure despite conventional sequential embryo transfer, what alternative strategies should be considered for subsequent transfer to target the WOI and optimize embryo–endometrium interactions? In this study, we present a case series of three patients with a history of recurrent implantation failure who achieved positive clinical pregnancy with sequential blastocyst transfer.

### 1.1 Case presentation

RIF should moved from a population-level evaluation to a individual-level diagnosis, a threshold of >60% cumulative chance of pregnancy is recommended to guide the clinical decision on whether to simply proceed to a further transfer or investigations should be employed to figure out the contributing factors (6). According to an estimating model, RIF is defined as three or more failed embryo transfer cycles in women under 40 years of age with embryos of unknown euploidy. Taking into account the pregnancy rate and embryo stage, some patients might have a >60% likelihood of implantation after two embryo transfers. Because the vast majority of patients were referred to our hospital from distant locations, investigations recommended by the ESHRE working group (6) were generally initiated after two failed transfer cycles to help prevent further implantation failure. This workup included reassessment of lifestyle factors, three-dimensional ultrasound screening, hysteroscopy, chronic endometritis (CE) evaluation, and assessment of thyroid function, glycolipid metabolism, antiphospholipid syndrome (APS), thrombophilia, and other suspicious immunological risk factors. Apart from insulin resistance, mild CE, and intrauterine adhesion (IUA), which were routinely treated before ET, there were no obvious underlying disorders identified. Endometrial thickness exceeded 7 mm before ET in all transfer cycles and all transferred embryos were derived from IVF cycles with unknown euploidy.

Herein, we discuss a different transfer approach for three consecutive patients with unexplained RIF and primary infertility to explore its potential benefits after implantation failure with conventional sequential cleavage/blastocyst transfer. A total of five patients underwent sequential blastocyst transfer at Hainan Kapok Hospital from March 2025 to October 2025. Two patients were excluded: One had experienced three implantation failures and achieved clinical pregnancy but had not received prior conventional sequential transfer, as only blastocysts had been cryopreserved; the other had experienced an early miscarriage and a chemical pregnancy among her four unsuccessful embryo transfer attempts, which did not meet the criteria for RIF.

## 2 Case 1

A 28-year-old woman presented to our hospital in August 2024 with a 5-year history of primary infertility. Fallopian tube obstruction was confirmed through infertility workup, and laparoscopic surgery was recommended to reconstruct the tube. However, attempts at natural conception failed in the following year. The couple subsequently undertook one complete IVF cycle in another reproductive center. The long protocol was applied for ovarian stimulation and three good-quality cleavage embryos were cryopreserved. Diagnostic hysteroscopy prior to embryo transfer found no abnormalities. The endometrium and cavity were normal. With the endometrium prepared in a natural cycle, two separate frozen embryo transfer (FET) cycles resulted in negative pregnancy tests.

In our hospital, ovarian stimulation was performed with a Progestin-Primed Ovarian Stimulation protocol. A total of two cleavage embryos and eleven blastocysts were harvested and cryopreserved. The patient reported normal findings of antiphospholipid antibody (APA), thyroid function, thrombophilia screening, immunological markers, and transvaginal sonography (TVS). An insulin-releasing test found elevated plasma insulin levels at 1 and 2 h after glucose intake, and the oral glucose tolerance test (OGTT) was normal. Lifestyle modifications were adopted and inositol supplementation was initiated to improve insulin resistance.

In September 2024, moderate uterine adhesions were identified and treated under hysteroscopy. At the same time, histopathological evaluation of an endometrial biopsy reported no evidence of CE (0 CD138-positive plasma cells). Karyotype analysis of the couple returned normal results. Before embryo transfer, a second hysteroscopy confirmed a normal uterine cavity. The endometrial preparation was underwent with a gonadotropin-releasing hormone (GnRH) agonist-suppressed hormone replacement therapy (HRT) cycle and one good-quality blastocyst (D5,4AA) was transferred, but the cycle failed. In the following transfer cycle, endometrial preparation was conducted with ovarian stimulation and a conventional sequential embryo transfer was performed: one cleavage-stage embryo (8C/I) and one blastocyst (D5,4AA). However, implantation was unsuccessful again. The HRT protocol used at our center is as follows: Estradiol is administered in a step-up protocol from 3–4 to 6–8 mg/day for at least 10 days; once the endometrial thickness reaches ≥7 mm, progesterone is supplemented with a combination of oral dydrogesterone 30 mg/day, subcutaneous progesterone 25 mg/day, and vaginal micronized progesterone (Utrogestan) 600 mg/day. Luteal-phase support follows the same progesterone supplementation, and the dosage is increased for rescue when the progesterone level is <10 ng/mL (17).

For this patient, sequential blastocyst transfer was adopted following HRT endometrial preparation: One blastocyst (D5,4BB) was transferred on Day 5 of progesterone supplementation and another blastocyst (D6,4BB) was transferred on Day 6. A positive pregnancy was confirmed and a healthy baby girl weighing 2800 g was delivered vaginally at 39 weeks of gestation, without pregnancy, perinatal, and neonatal complications.

## 3 Case 2

Diagnosed with primary infertility due to fallopian tube obstruction at the age of 31 in 2021, this patient was asked to consider IVF treatment at another reproductive center. Two D5 blastocysts were obtained from a GnRH antagonist stimulation cycle and transferred in two separate frozen embryo transfer cycles, both of which failed to implant. Based on her age, the number and stage embryos transferred, her clinical prognosis, and the center-specific pregnancy rate per transfer, the estimated cumulative probability of implantation after two embryo transfers exceeded 60%.

In July 2024, the patient was referred to our hospital for further ART. Undergoing ovarian stimulation with a long protocol, two cleavage-stage embryos and three blastocysts were yielded and cryopreserved. Prior to embryo transfer, RIF-related investigations were undertaken: Transvaginal ultrasound screening was normal. The antiphospholipid antibody profile [anticardiolipin antibody (ACA), anti-beta-2 glycoprotein I antibodies (αβ2GPI), lupus anticoagulant (LA), anti-phosphatidylserine/prothrombin antibodies (aPS/PT)], thrombophilia panel screening (coagulation function, protein S, protein C and antithrombin Ⅲ), immunological marker panel (ANA profile, IL-10, TNF-α), and parental karyotyping were all negative. Diagnostic hysteroscopy revealed a normal cavity. Endometrial biopsy was performed after hysteroscopy for histological and immunohistochemistry (IHC) evaluation, which identified six CD138/MUM-1-positive plasma cells per high-power field (HPF) (normal reference: <5/HPF). The patient received oral doxycycline 100 mg and ornidazole 500 mg twice daily for 14 days. Cure was not evaluated after treatment because of the mild nature of CE and a cure rate of approximately 90% with a 14-day course of oral antibiotic therapy (18).

Conventional sequential ET with one D3 embryo (8/I) and one D5 blastocyst (4BA) was performed in a GnRH agonist downregulated HRT cycle, ending in a biochemical pregnancy in February 2025. Subsequently, endometrial preparation was performed in a mild ovarian stimulation cycle with letrozole 5 mg/day for 5 days plus HMG 37.5 IU daily for 2 days. Triggering was performed with 10,000 IU human chorionic gonadotropin (hCG), and luteal-phase support consisted of dydrogesterone 10 mg twice daily and subcutaneous progesterone 25 mg/day. Sequential blastocyst transfer was employed. One blastocyst (D6,4BB) was transferred on Day 7 after hCG trigger and another blastocyst (D6,4CB) on Day 8. A positive hCG test was confirmed and a twin pregnancy was identified subsequently. One fetus vanished spontaneously around 12 weeks of gestation. A healthy baby girl weighing 2,900 g was delivered via cesarean section at 39 weeks with no maternal and perinatal complications.

## 4 Case 3

The third case involved a 39-year-old woman with a 5-year history of primary infertility. She had previously undergone one fresh embryo transfer cycle and two FET cycles with HRT and GnRHa + HRT for endometrial preparation consecutively at a local reproductive center in 2023 and 2024. All three ET cycles resulted in implantation failure. After the first embryo transfer, hydrosalpinx was discovered by transvaginal ultrasound and she underwent laparoscopic bilateral proximal tubal occlusion by electrocautery combined with distal salpingostomy in July 2024.

Her anti-Müllerian hormone (AMH) level was 1.46 ng/mL when she came to our clinic in 2025. Apart from moderate uterine adhesion—identified and treated under hysteroscopy followed by normal hysteroscopic evaluation—all RIF-related investigations were negative, consistent with findings in Cases 1 and 2. The workup included karyotyping of the couple, APS screening, thrombophilia panel, thyroid function, glycolipid metabolism, autoimmune antibodies, chronic endometritis evaluation, and peripheral immune cell profiling.

In April 2025, ovarian stimulation was carried out using a GnRH agonist long protocol, harvesting one cleavage-stage embryo and four blastocysts. A fresh sequential embryo transfer was performed with one cleavage-stage embryo (8C/I) transferred on Day 3 and one blastocyst (D5,4BB) transferred on Day 5 after oocyte pickup. However, implantation did not occur. Three remaining blastocysts were cryopreserved.

In the following ET cycle, sequential blastocyst embryo transfer was implemented on Day 7 (D5,3BB) and Day 8 (D5,3BC) after hCG triggering in a mild ovarian stimulation cycle. A heartbeat clinical pregnancy was confirmed, but unfortunately ended in spontaneous miscarriage at 8 weeks of gestation. Genetic analysis of pregnancy tissue using next-generation sequencing combined with whole-genome SNP Chip (Yikon Genomics Co., Ltd.. Suzhou, Jiangsu, China) identified a pathogenic 32.70 Mb deletion of chromosome 21.

In the subsequent month, an additional FET cycle was performed with the last remaining blastocyst (D5,4CB) in a modified natural cycle, with luteal-phase support consisting of dydrogesterone 10 mg three times a day. The patient achieved successful clinical pregnancy and smoothly proceeded to 38 weeks of gestation without pregnancy complications. Her expected date of delivery is 13 September 2026. Followup: A healthy baby boy weighing 3250 g was delivered via cesarean section at 38^+4^ weeks with no maternal and perinatal complications.

## 5 Discussion

This case series described a sequential blastocyst transfer for IVF/intracytoplasmic sperm injection (ICSI) patients with unexplained recurrent implantation failure, in which two blastocysts were transferred on two consecutive days within a frozen embryo transfer cycle. All three patients achieved positive clinical pregnancies after multiple prior implantation failures. Two women successfully delivered healthy babies, while one woman of advanced maternal age experienced a miscarriage after which genetic analysis of the pregnancy tissue identified a pathogenic 32.70 Mb deletion of chromosome 21.

RIF is a secondary clinical phenomenon within ART characterized by multifactorial and heterogeneous etiology (19). In general, potential causes are evaluated from three aspects: maternal, paternal, and embryonic factors. For patients with available frozen embryos, female factors are considered the most relevant. In accordance with ESHRE recommendation, appropriate RIF investigations were performed prior to further embryo transfer. No obvious causative factors were identified and embryo implantation still failed following conventional sequential cleavage/blastocyst ET.

Therefore, we focused on the WOI as a suspected contributing factor (11). The WOI is a time frame during which the endometrium is well prepared to receive the embryo to adhere and implant. Various WOI assessment and diagnostic tools have been proposed and developed (20–22), including ultrasonographic evaluation of endometrial receptivity, histological endometrial dating, pinopode assessment by scanning electron microscopy, and the endometrial receptivity array (ERA). A growing body of evidence indicates that WOI displacement occurs in a substantial proportion of RIF patients. The incidence of WOI displacement has been reported in up to 50%–60% of RIF patients (23) and delayed receptivity has been observed in up to 97% of cases involving a displaced WOI (22). Personalized embryo transfer (pET) (24) guided by endometrial receptivity analysis has been utilized to optimize clinical outcomes. However, current endometrial receptivity analysis tools are invasive, time-consuming, costly, and subject to variability. Moreover, the effectiveness of analysis-guided pET remains controversial and requires further investigation (11, 25). After detailed consultation, the patients declined ERA and requested to proceed with the next embryo transfer.

Sequential ET was first introduced by Abramovici in 1988, when there was no embryo freezing (26). Conventionally, sequential ET involves a two-step transfer of a cleavage-stage embryo and a blastocyst within a single transfer cycle. Most studies have demonstrated that sequential ET improves implantation rates and clinical pregnancy rates compared with routine cleavage embryo or blastocyst transfer in RIF patients (14, 15, 27). Some observational studies (14, 15) have further reported that sequential ET is associated with higher clinical pregnancy rates and lower miscarriage rates than double blastocyst transfer, without adversely affecting perinatal outcomes (28). Sequential ET is therefore considered an effective option for RIF patients.

All three patients in this case series had previously experienced failed conventional sequential embryo transfersas summarized in Tables 1–3. Given the high prevalence of delayed endometrial receptivity and the proposed beneficial mechanism of sequential ET for embryo implantation, we adopted a two-step blastocyst transfer approach for these patients, who achieved successful clinical pregnancy. Jin et al. (29) compared three strategies of frozen embryo transfer in RIF patients undergoing HRT cycles and found that the clinical pregnancy rate was higher with sequential blastocyst transfer on Day 5 and Day 6 than with traditional sequential FET. However, that study had potential bias as the patients in the sequential D3/D5 FET group were significantly older, had significantly lower AMH levels, and had experienced more previous failed cycles. Moreover, the strategy for embryo culture and freezing at our center dictates that at least two good-quality cleavage-stage embryos are cryopreserved first and the remaining ones are cultured to blastocyst. Taking available evidence and safety into consideration, conventional sequential cleavage/blastocyst transfer should be considered first for RIF patients and sequential blastocyst embryo transfer may serve as an alternative for the following ET cycles.

**Table 1:** The summary of transfer cycles in Case 1.

ET cycle	Transfer date	Age at ET (years)	Type of embryo transfer	Endometrial preparation protocol	Stage and grade of embryos transferred	Number of embryos transferred	Timing of embryo transfer	Outcome
1st	February 2024	27	Frozen	Natural	D3; 8/I	1	LH + 4	Implantation failure
2nd	April 2024	27	Frozen	Natural	D3; 8/I,7/II	2	LH + 4	Implantation failure
3rd	November 2024	28	Frozen	GnRHa + HRT	D5; 4AA	1	P + 5	Implantation failure
4th	December 2024	28	Frozen	OI	Sequential transfer D3; 8/I D5; 4AA	2	hCG + 5/hCG + 7	Implantation failure
5th	March 2025	28	Frozen	HRT	Sequential transfer Day 5: D5; 4BB Day 6: D6; 4BB	2	P + 5/P + 6	Single live birth

ET, embryo transfer; HRT, hormone replacement therapy; OI, ovulation induction; LH, luteinizing hormone; hCG, human chorionic gonadotropin; P, progesterone.

**Table 2:** The summary of transfer cycles in Case 2.

ET cycle	Transfer date	Age at ET (years)	Type of embryo transfer	Endometrial preparation protocol	Stage and grade of embryos transferred	Number of embryos transferred	Timing of embryo transfer	Outcome
1st	July 2021	30	Frozen	HRT	D5; 4BC	1	P + 5	Implantation failure
2nd	August 2021	31	Frozen	Natural	D5; 4BC	1	LH + 6	Implantation failure
3rd	February 2025	35	Frozen	GnRHa + HRT	Sequential transfer D3; 8/I D5; 4BA	2	P + 3/P + 5	Biochemical pregnancy
4th	May 2025	35	Frozen	OI	Sequential transfer D6; 4BB D6; 4CB	2	hCG + 7/hCG + 8	Single live birth

ET, embryo transfer; HRT, hormone replacement therapy; OI, ovulation induction; LH, luteinizing hormone; hCG, human chorionic gonadotropin; P, progesterone.

**Table 3:** The summary of transfer cycles in Case 3.

ET cycle	Transfer date	Age at ET (years)	Type of embryo transfer	Endometrial preparation protocol	Stage and grade of embryos transferred	Number of embryos transferred	Timing of embryo transfer	Outcome
1st	March 2023	36	Fresh	—	D3; 8/II	1	OPU + 3	Implantation failure
2nd	September 2024	37	Frozen	HRT	D3; 7/IIa	2	P + 3	Implantation failure
3rd	December 2024	38	Frozen	GnRHa + HRT	D3; 7/IIb D3; Ⅲ	2	P + 3	Implantation failure
4th	April 2025	39	Fresh	—	Sequential transfer D3; 8/I D5; 4BB	2	P + 3/P + 5	Implantation failure
5th	September 2025	39	Frozen	OI	Sequential transfer D5; 3BB D5; 3BC	2	hCG + 7/hCG + 8	Early miscarriage
6th	December 2025	39	Frozen	Modified Natural	D5; 4CB	1	hCG + 7	single live birth

ET, embryo transfer; HRT, hormone replacement therapy; OI, ovulation induction; hCG, human chorionic gonadotropin; P, progesterone.

The potential mechanisms underlying the improved implantation outcomes of sequential ET involve several aspects (15, 29). First, endometrial microstimulation by the transfer catheter during the first transfer may trigger and activate molecular mediators of endometrial receptivity. Second, two-interval embryo transfer may alter and match the displaced WOI. In addition, the embryo transferred earlier is thought to promote endometrial receptivity and embryo–endometrium interaction by secreting embryo-derived factors and co-culturing with the endometrium.

Moreover, endometrial preparation is an important factor influencing frozen embryo transfer outcomes. The most common approaches include natural cycle with or without hCG triggering, ovulation induction, and HRT with or without GnRH agonist suppression. There is insufficient evidence to support the superiority of one regimen over another (30), and the same applies to patients with unexplained RIF (31). At the same time, individualized luteal-phase support in HRT cycles has become standard clinical practice (32). Therefore, individualized treatment based on patient and cycle characteristics is crucial (33). Reproductive physicians operate under heavy psychological pressure and aim to help patients achieve pregnancy efficiently and cost-effectively, rather than simply offering repeated embryo transfers.

The prevalence of multiple pregnancy (MP) is higher in ART and is primarily associated with the number and stage of transferred embryos. Selective single-embryo transfer is widely advocated worldwide to reduce the incidence of multiple pregnancy. In our hospital, patients with a good prognosis are strongly recommended to employ a single-embryo transfer regardless of the embryo stage. According to Chinese expert consensus, no more than two embryos are recommended per transfer cycle, regardless of age and the number of previous transfer cycles. For patients with a poor prognosis, including RIF, transfer of two embryos is often recommended, taking into account patient age, embryo stage and quality, embryo ploidy, and ART history (34). Our three patients were counseled in detail regarding the risk of multiple pregnancy from an additional embryo, and written informed consents were obtained. Of the three cases, one patient had a vanishing twin, which was a potential risk of MP. Existing evidence shows that the prevalence of multiple pregnancy in sequential cleavage/blastocyst ET is significantly lower than that in double cleavage or blastocyst transfer (15). Similarly, the multiple pregnancy rate with sequential blastocyst ET is considerably lower than that with double blastocyst transfer (29). Impaired endometrial receptivity is considered an underlying cause of unexplained RIF, and the potential of implantation per embryo transferred may be relatively lower. In this context, some embryos may only play a role in promoting endometrial receptivity rather than implanting themselves. The risk of multiple pregnancy and adverse perinatal outcomes with sequential blastocyst transfer should be investigated through good-quality studies in the future.

Case series occupy the lowest tier with the evidence hierarchy. Observations derived from a small number of cases, without a control group or systematic methodology, cannot be interpreted as evidence of effectiveness or generalized to the broader population of unexplained RIF patients. The observations from this case series may help generate questions and hypotheses for future studies. In addition, bias and confounding factors—such as endometrial preparation regimen, the number and grade of embryo transferred, and the impact of repeated transfers—should be adequately controlled. Furthermore, state-of-the-art endometrial receptivity assessment should be considered prior to embryo transfer to investigate the hypothesis that the WOI is displaced in patients with unexplained RIF. Moreover, preimplantation genetic testing for aneuploidy (PGT-A) should be considered for patients with RIF as an alternative strategy, as it has been shown to improve the positive serum β-hCG rate and ongoing pregnancy rate, particularly in RIF patients of advanced maternal age (35). Finally, the cumulative pregnancy rate may be considered the primary outcome.

## 6 Conclusion

In conclusion, unexplained RIF is a deeply distressing and condition for affected patients. There is no “one-size-fits-all” protocol for patients with repeated embryo transfer failure. Sequential blastocyst transfer may be a viable option for patients with unexplained RIF, particularly after implantation failure following conventional sequential cleavage/blastocyst ET. However, this case series is preliminary and hypothesis-generating. Well-designed comparative studies are warranted to evaluate the efficacy and safety of sequential blastocyst transfer in RIF patients.

## 7 Patient perspective

We have spent many years pursuing treatments for infertility. Repeated implantation failure is a deeply distressing and painful experience for us and our families. We have faced enormous psychological distress and financial burdens during the process, even facing the risk of unemployment and marital crisis. Every day, we dreamed of seeing a gestational sac and a lovely heartbeat on the screen in the ultrasound room. We were so grateful and extremely excited when our dream came true after the transfer. We do not know how it happened, and we are happy to share our experience for reference to others.

## Data Availability

The raw data supporting the conclusions of this article will be made available by the authors without undue reservation.
